# IPEC-J2 Autophagy Induced by TLR4 and NSP6 Interactions Facilitate Porcine Epidemic Diarrhea Virus Replication

**DOI:** 10.3390/v16111787

**Published:** 2024-11-17

**Authors:** Haiyuan Zhao, Dianzhong Zheng, Qinyuan Chang, Hailin Zhang, Yilan Shao, Jiaxuan Li, Wen Cui, Yanping Jiang, Lijie Tang, Yijing Li, Xiaona Wang

**Affiliations:** 1 College of Veterinary Medicine, Northeast Agricultural University, Harbin 150030, China; zhywxn1925@163.com (H.Z.); changqinyuan1017@163.com (Q.C.); shaoyilan232@163.com (Y.S.); lijiaxuan.1993@163.com (J.L.); cuiwen_200@163.com (W.C.); jiangyanping8198@163.com (Y.J.); 2 Institute of Animal Husbandry, Heilongjiang Academy of Agricultural Sciences, Harbin 150086, China; dianzhongzheng11@163.com; 3 Chongqing Academy of Animal Science, Chongqing 402460, China; zhanghl0523@163.com; 4 Heilongjiang Key Laboratory for Animal Disease Control and Pharmaceutical Development, Harbin 150030, China

**Keywords:** PEDV NSP6, IPEC-J2, TLR4, AKT-mTOR, autophagy, replication

## Abstract

Autophagy is an important cellular response against intracellular pathogens. However, some viruses have evolved mechanisms to hijack this defensive process to provide favorable conditions for virus replication in host cells. The porcine epidemic diarrhea virus (PEDV) has been shown to alter autophagy pathways; however, it is still unknown through which receptors PEDV induces autophagy in IPEC-J2 cells, whether autophagy facilitates PEDV replication, and which functional domains of PEDV proteins are primarily responsible for inducing autophagy. Here, we found that PEDV infection induces autophagy in host cells via distinct and uncoupled molecular pathways. RNA-seq technology was used to analyze the expression patterns of intracellular genes in PEDV-infected IPEC-J2 cells using transcriptomics. The results demonstrate that PEDV triggers autophagy via the cellular pathogen receptor TLR4 and the AKT-mTOR pathway. As evidenced by autophagosome detection, PEDV infection increases autophagosomes and light chain 3 (LC3)-II as well as downregulated AKT-mTOR phosphorylation. Our study revealed that the binding of the viral protein NSP61-2C (56-151aa) to TLR4 triggers autophagy and inactivates the AKT-mTOR pathway, both of which are critical for facilitating PEDV infection. Through screening and analysis, TLR4 was found to be a key gene involved in PEDV-induced autophagy. The screening of the key functional domains of NSP6 (56-151aa) for their ability to induce autophagy in IPEC-J2 cells provided a basis for the in-depth analysis of the pathogenic mechanism of PEDV infection-induced autophagy and promotion of self-replication and also provided an important target for the study of PEDV antiviral drugs. In conclusion, we elucidated that the PEDV infection of IPEC-J2 cells could induce autophagy and found that PEDV could use autophagy to promote its own replication.

## 1. Introduction

The porcine epidemic diarrhea virus (PEDV) is a highly pathogenic, enteric coronavirus that is transmissible in swine. PEDV infection can result in severe diarrhea, dehydration, and death. Neonatal piglets exhibit the highest mortality rates, which causes serious economic losses in the global swine industry [1,2,3]. PEDV is an enveloped, single-stranded, forward RNA virus with a genome size of approximately 28 kb [4]. The PEDV genome encodes the following: four structural proteins (spike protein [S], envelope protein [E], membrane protein [M], nucleocapsid protein [N]), sixteen non-structural proteins (NSP1–NSP16), and one auxiliary protein (ORF3) [5]. Each structural and non-structural protein of PEDV plays an important role in virus replication, transcription, translation, and host interactions. Researchers have developed vaccines and antiviral drugs against PEDV [6,7]. Non-structural proteins (NSPs) are key elements of the replication and transcription complex of coronaviruses and can evade detection by the immune system. Among the 16 NSPs of coronaviruses, non-structural protein 6 (NSP6) has been shown to induce the formation of autophagosomes in the endoplasmic reticulum of host cells [8]. NSP6 expression in the avian coronavirus infectious bronchitis virus (IBV) can increase intracellular LC3 (microtubule-associated proteins 1A/1B, light chain 3B) levels [9]. Currently, autophagy has been shown to be beneficial to PEDV replication, but the mechanism is unknown, and few studies think it may be related to the expression of inflammatory cytokines and positive feedback to the NF-κB signaling pathway during PEDV infection [10]. Rapamycin-induced autophagy was found to inhibit PEDV infection and reduce PEDV-induced epithelial cell death [11]. A previous study found that NSP6 played a key role in inducing autophagy [12].

PEDV can infect and rapidly replicate in the epithelial cells of the small intestinal villi, resulting in the destruction of a large number of intestinal cells, the atrophy of the intestinal villi, the malabsorption of nutrients, and diarrhea in piglets [13,14]. PEDV primarily targets porcine intestinal epithelial cells (IECs); however, most studies on PEDV have used African monkey kidney epithelial cell lines such as Vero-E6 cells [10,15]. PEDV has strong tropism for porcine intestinal tissue. After entering the host, PEDV preferentially infects the jejunum and ileum, with a small amount infecting the duodenum [16].

It has been observed that certain viruses closely interact with specific pathways that typically regulate infections [17,18]. Viral entry relies on its capacity to bind to specific cellular receptors that facilitate invasion, such as Toll-like receptors (TLRs). Hepatitis B virus-induced autophagy promotes liver cancer progression via TLR4 [19,20,21,22]. The Avian Influenza Virus (AIV) utilizes TLR4 to initiate signaling pathways that promote the autophagic degradation of viral particles, which is a process that paradoxically enhances viral replication while suppressing effective immune responses [23]. Moreover, the Feline Infectious Peritonitis Virus (FIPV) has been shown to activate TLR4-mediated autophagy to sustain its replication within feline macrophages, thus evading the host’s innate immune response and prolonging the infection [24]. However, it is still unclear through which receptors PEDV induces autophagy in IPEC-J2, whether autophagy is beneficial for PEDV replication, and the main functional domains of PEDV protein-induced autophagy.

Autophagy, also known as cellular self-digestion, is an evolutionarily conserved cellular process that degrades senescent proteins, causes damage to organelles, and invades pathogens through lysosomes [25,26]. Autophagy is a powerful catabolic pathway, and as such, it is activated during viral infections to degrade viruses that invade host cells [27]. However, an increasing number of studies have found that many viruses have evolved strategies to maintain their life cycle and pathogenicity by hijacking and disrupting autophagy signaling in host cells [28,29]. Research has demonstrated that coronavirus infection is linked to the autophagic process, with coronavirus non-structural proteins promoting autophagosome formation from the endoplasmic reticulum through an omegasome intermediate [30].

The mTOR kinase-dependent signaling pathway has been implicated in autophagy mediation [31]. A study found that the HSP90AA1 receptor interacted with the AKT-mTOR pathway to trigger autophagy [32]. The suppression of the AKT-mTOR signaling pathway also influenced autophagic production induced by viruses, such as coxsackievirus B3 and foot-and-mouth disease viruses [33,34]. Hence, a close connection exists between autophagy and the AKT-mTOR signaling pathway during viral infection. In this study, we aimed to uncover the mechanisms by which PEDV and the functional domains of its NSP6 protein induce autophagy during infection and how PEDV leverages the host cell’s autophagic machinery to enhance replication. This research provides a theoretical foundation for understanding PEDV pathogenesis and developing strategies to control viral infection.

## 2. Materials and Methods

### 2.1. Cell Lines and Virus Stock

The intestinal porcine epithelial cell line-J2 (IPEC-J2) was obtained from our laboratory. The cells were cultured in Dulbecco’s Modified Eagle Medium (DMEM) (LongGene Scientific Instruments Co., Ltd.; Hangzhou, China) supplemented with 10% fetal bovine serum (Gibco, Thermo Fisher Scientific Inc., Waltham, MA, USA), 100 IU/mL penicillin, and 10 μg/mL streptomycin (Thermo Fisher Scientific Inc.) at 37 °C in 5% CO_2_.

This study was approved by the Institutional Committee of the Northeast Agricultural University, with the approval number [NEAUEC20210305]. The virulent PEDV CH/HLJ/18 strain (GenBank accession number MW561264.1) was obtained from our laboratory. Viral stock was prepared by collecting the supernatants from infected cells once a cytopathic effect (CPE) was visible in approximately 80% of the cell population. To measure viral titers, IPEC-J2 cells were seeded in 96-well plates and inoculated with 10-fold serial dilutions of the virus. The final concentration of trypsin was 25 μg/mL, and the solution was discarded after being incubated for 1 h in a 5% CO_2_ incubator at 37 °C. Cells were then placed in a DMEM virus maintenance solution with a final concentration of 6 μg/mL trypsin. Meanwhile, control IPEC-J2 cells were cultured in an incubator at 37 °C and 5% CO_2_ for 48 h. The virus TCID_50_ was determined according to the Reed Muench method [35].

### 2.2. Antibodies, Plasmids and Reagents

The mouse anti-PEDV-N monoclonal antibody was produced by our laboratory [36], the mCherry-GFP-LC3 carrier (Heyuan Biotechnology Co., Ltd., Shanghai, China), and the pCMV-HA eukaryotic expression vector were stored in our laboratory. The rabbit anti-porcine p62 polyclonal antibody (ABclonal Technology, A11247; Woburn, MA, USA), rabbit anti-porcine LC3 polyclonal antibody (Affinity Biosciences, AF4650; Cincinnati, OH, USA), rabbit anti-porcine TLR4 polyclonal antibody (ABclonal, A11226), rabbit anti-porcine AKT polyclonal antibody (ABclonal, A18120), rabbit anti-porcine p-AKT polyclonal antibody (ABclonal, AP1068), rabbit anti-porcine mTOR polyclonal antibody (ABclonal, A2445), rabbit anti-porcine p-mTOR polyclonal antibody (ABclonal, AP0490), rabbit anti-ACTB (Thermo Fisher Scientific, MA5-42946), HRP-conjugated goat anti-rabbit IgG (Zhongshan Golden Bridge Biotechnology Co., Ltd., ZB-2301; Beijing, China), FITC-conjugated goat anti-rabbit IgG (Zhongshan Golden Bridge Biotechnology Co., Ltd., ZF-0311), HRP-conjugated goat anti-mouse IgG antibody (Zhongshan Golden Bridge Biotechnology Co., Ltd., ZB-2305), and TRITC-conjugated goat anti-mouse IgG antibody (Zhongshan Golden Bridge Biotechnology Co., Ltd., ZF-0315) were used in our study. Porcine insulin (Yuanye Bio-Technology Co., Ltd., S24703; Shanghai, China), chloroquine (Sigma Pharmaceuticals, C6628; North Liberty, IA, USA), rapamycin (Sigma Pharmaceuticals, V900930), and the Lipofectamine 3000 transfection reagent (Thermo Fisher Scientific) were purchased for this study.

To identify the key functional domains of Nsp6 responsible for inducing autophagy in cells, we first analyzed the gene sequence of Nsp6 using the SMART website to predict potential functional domains and truncated Nsp6 and constructed the eukaryotic expression plasmids pCMV-Nsp6-1, pCMV-Nsp6-2, and pCMV-Nsp6-3. To further identify the key functional domains of Nsp6 responsible for inducing autophagy in cells, we analyzed the gene sequences of Nsp6-1 and Nsp6-2 using the SMART website to predict potential functional domains and truncated Nsp6-1 and Nsp6-2 and constructed the three eukaryotic expression vectors pCMV-Nsp61A, pCMV-Nsp61B, and pCMV-Nsp61-2C.

### 2.3. Viral Infection and Cell Treatment

IPEC-J2 cells were infected with PEDV at a multiplicity of infection (MOI) of 1 in DMEM at 37 °C for the specified durations, with uninfected cells serving as controls. To assess autophagy activation and inhibition, cells were pretreated with rapamycin (50 nM) for 4 h or insulin [37] (2 μM) for 6 h, respectively, before viral infection. For experiments aimed at inhibiting the fusion of autophagosomes and lysosomes, the IPEC-J2 cells were treated with chloroquine (60 μM) and infected with PEDV for 4 h [38]. For the viral protein stimulation experiment, the IPEC-J2 cells were transfected with pCMV-NSP6, pCMV-NSP6-1, pCMV-NSP6-2, pCMV-NSP6-3, pCMV-NSP61A, pCMV-NSP61B, and pCMV-NSP61-2C for 24 h at 37 °C.

### 2.4. Relative Expression Analysis of PEDV-N Gene

To analyze PEDV-N mRNA levels, total cellular RNA was extracted from IPEC-J2 cells, and cDNA was synthesized using RNA reverse transcriptase and oligo (dT) primers (TaKaRa, Dalian, China) following previously established protocols [39]. The expression of the target gene was calculated using the 2^−ΔΔCt^ method [40]. The primers are listed in Table 1.

### 2.5. Transfection and Gene Silencing with siRNAs

SMARTpool siRNAs targeting TLR4, the transferrin receptor (TFRC), and gamma-aminobutyric acid type A receptor subunit gamma3 (GABRG3) were designed and synthesized using an external siRNA service (GenePharma Biotechnology, Shanghai, China). For each gene, the three target sequences with the highest scores were selected, and the most effective siRNA was chosen (Table 2). IPEC-J2 cells were cultured to 80% confluence in 6-well plates and transfected with 1 μg of plasmid or 50 nM siRNA per well using the TurboFect transfection reagent (Thermo Fisher Scientific, L3000015), following previously established methods [41,42]. The cells were then incubated in a fresh medium until either harvested or until the culture medium was collected at designated time points. A non-targeting siRNA was used as the negative control. Silencing efficiency was assessed by RT-qPCR.

### 2.6. Western Blotting Analysis

IPEC-J2 cell samples were processed at increasing time intervals following infection or transfection. The protein composition of the cells over time was assessed using immunoblotting with primary antibodies against cellular proteins. At designated time points, cell lysates were prepared, followed by boiling the samples for 10 min. The proteins were separated by SDS-PAGE and transferred onto 0.22 μm polyvinylidene difluoride (PVDF) membranes (Millipore, Milford, MA, USA), using a wet transfer method. The membranes were blocked with 5% skim milk at 37 °C for 1 h and incubated with primary antibodies, followed by HRP-conjugated secondary antibodies for 12 h at 4 °C. Bound antibodies were visualized using ECL detection reagents (Thermo Scientific, 32209). Images were captured with a scanner (Thermo Scientific), and the staining intensity of target proteins was quantified using ImageJ software (NIH, lmageJ 1.53t, Java 1.8.0 345 (64-bit)). All target proteins and internal loading controls were confirmed to be within the same linear detection range.

### 2.7. TEM Sample Preparation and Analysis

IPEC-J2 cells were cultured in 6-well plates, and the cell density was adjusted to 2 × 10^6^ cells/well. PEDV infection was carried out after the cells grew to 80% confluency; non-infected IPEC-J2 cells were used as the negative control group. PEDV-infected cells were incubated for 18 h. Then, the supernatant was discarded, and 1 mL sterile PBS was added to allow the removal of cells using a cell scraper. The resulting cell suspension was transferred to a 1.5 mL sterile EP tube for TEM sample preparation as follows. The suspension was centrifuged at 1000 rpm/min for 3 min, and the supernatant was discarded; a 2.5% glutaraldehyde fixative was added and fixed at room temperature for 3 h, after which the fixative was discarded. The cells were then washed twice with PBS. The cells were fixed with 1% osmic acid at 4 °C for 2 h, and with this, the fixing solution was discarded. The cells were then washed twice with phosphate-buffered saline (PBS). Subsequently, 50%, 70%, 90%, and 100% ethanol, a mixture of 100% ethanol and 100% acetone (1:1), and 100% acetone were used for dehydration. A 100% acetone and embedding solution (1:1) was then added for soaking. After embedding, polymerization, and block repair, an ultrathin microtome was used to slice sections from the prepared sample block. These sections were double-stained with uranyl acetate and lead citrate and were finally observed using a transmission electron microscope.

### 2.8. Immunofluorescence Microscopy

Following the indicated treatments, mCherry-GFP-LC3 adenovirus vector-infected IPEC-J2 cells were infected with PEDV for 18 h. pCMV-NSP6, pCMV-NSP6-1, pCMV-NSP6-2, pCMV-NSP6-3, pCMV-NSP61A, pCMV-NSP61B, and pCMV-NSP61-2C IPEC-J2 cells were infected with the mCherry-GFP-LC3 adenovirus vector and analyzed under a fluorescence microscope (Bio-Rad, Hercules, CA, USA).

For indirect immunofluorescence experiments, pCMV-NSP61-2C IPEC-J2 cells were washed 3 times with PBS and fixed in 4% paraformaldehyde. The cells were washed three times with PBS and treated with 0.2% Triton X-100 (Sangon Biotech, A110694; Shanghai, China) for 10 min. Subsequently, the cells were blocked with 0.3% bovine serum albumin (BSA; Sigma, Ronkonkoma, NY, USA) for 30 min at 37 °C, followed by incubation with the appropriate primary antibodies for 1 h at 37 °C. After washing, the cells were incubated with FITC- or TRITC-conjugated secondary antibodies. Finally, the cells were rinsed three times with PBS and visualized under an immunofluorescence microscope (Bio-Rad).

### 2.9. Statistical Analysis

Data are presented as the mean ± standard deviation (SD). Differences between treatment groups were analyzed using one-way analysis of variance (ANOVA) followed by Tukey’s multiple comparisons test with GraphPad Prism software (version 5.0). A *p*-value of <0.05 was considered statistically significant.

## 3. Results

### 3.1. PEDV-Induced Autophagy Marker Production in IPEC-J2 Cells

**Figure 1:**
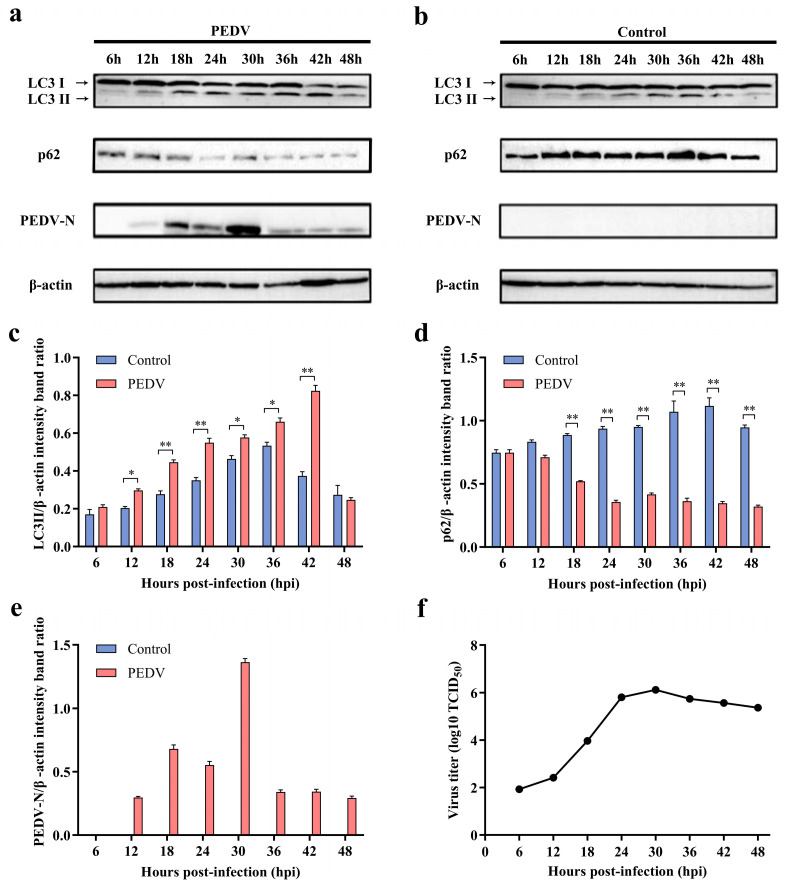
PEDV infection in IPEC-J2 cell-induced autophagy. (**a**,**b**) Western blot was used to detect changes in the expression of LC3-II, p62, and PEDV-N proteins in PEDV-infected IPEC-J2 cells at 6 h, 12 h, 18 h, 24 h, 30 h, 36 h, 42 h, and 48 h post-infection. Cell samples from non-infected cultures at the same time points were used as the control. (**c**) Quantitative analysis of LC3-II and β-actin. (**d**) Quantitative analysis of P62a and β-actin. (**e**) Quantitative analysis of PEDV-N and β-actin. (**f**) Determination of TCID_50_ of the PEDV. * (*p* < 0.05) and ** (*p* < 0.01).

LC3-II is widely recognized as a marker of autophagy [43]. In this study, we evaluated the autophagy response induced by PEDV infection over time in IPEC-J2 cells by measuring markers of autophagy at a multiplicity of infection (MOI) of one, using immunoblotting from 6 h to 48 h post-infection (hpi). The results indicated that PEDV infection induced significant autophagy (Figure 1a–d). Compared to that in control cells, the level of intracellular LC3-II in PEDV-infected IPEC-J2 cells obviously increased at 18 hpi (Figure 1a–c). In addition to LC3-II, we measured SQSTM1/p62 (sequestosome 1) protein levels as a target of autophagic degradation [43]. The results showed that p62 expression levels obviously decreased from 18 hpi to 48 hpi (Figure 1a,b,d), implying that an enhanced autophagic flux occurred at these time points following infection. Furthermore, the viral N protein was detectable at 12 hpi, and its levels rapidly increased at 18 hpi (Figure 1a,b,e). The viral titers also showed an upward trend from 6 to 30 hpi (Figure 1f). Therefore, for subsequent experiments, 18 hpi was considered the optimal time point for the evaluation of autophagy.

### 3.2. Observation of the Autophagosomes in IPEC-J2 Cells Using Transmission Electron Microscopy (TEM)

**Figure 2:**
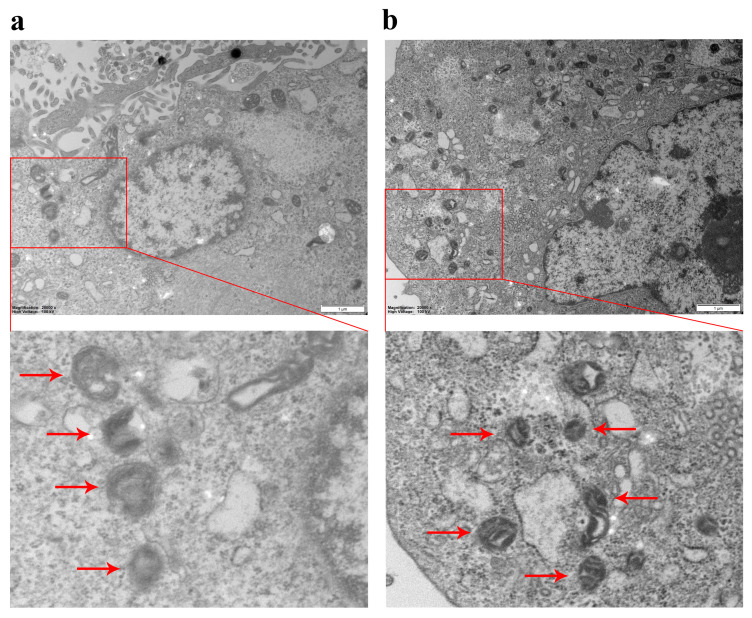
Autophagosome observed using TEM. (**a**) Cell control group (Scale, 1 μm) and magnified autophagosome structure in the control group (Scale, 500 nm); (**b**) PEDV-infected cells (Scale, 1 μm) and magnified autophagosome structure in the PEDV-infected group (Scale, 500 nm).

To determine whether PEDV infection regulated autophagy, TEM was used for the ultrastructural analysis of PEDV-infected IPEC-J2 cells (Figure 2). The results demonstrated that the number of double-membrane, autophagosome-like vesicles increased in the cytoplasm of PEDV-infected IPEC-J2 cells at 18 hpi (Figure 2b), whereas similar vesicles were rarely observed in the control IPEC-J2 cells (Figure 2a).

### 3.3. The Role of Autophagy in PEDV Replication

**Figure 3:**
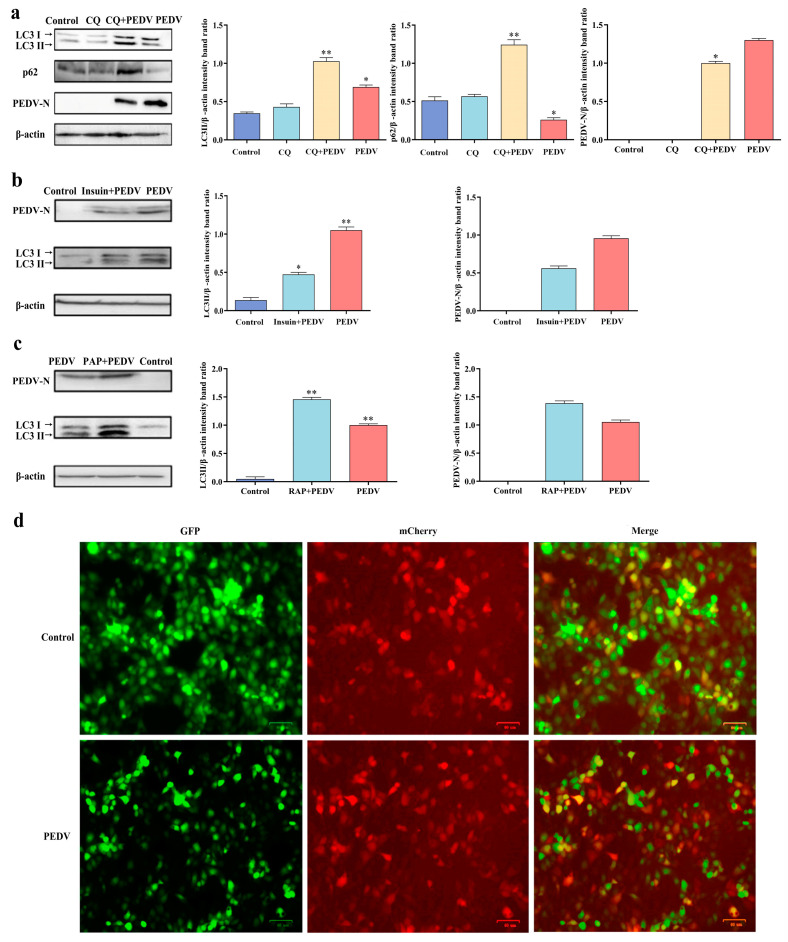
The role of autophagy in PEDV replication. (**a**) Expression levels of LC3-II and p62 in IPEC-J2 cells pretreated with different concentrations of chloroquine. (**b**) Expression level of LC3-II in IPEC-J2 cells treated with different concentrations of insulin. (**c**) Expression level of LC3-II in IPEC-J2 cells treated with different concentrations of rapamycin. The control group included normal cells. (**d**) The autophagic flow was detected using GFP-mCherry-LC3 dual fluorescence labeling. * (*p* < 0.05) and ** (*p* < 0.01).

Chloroquine has been demonstrated to prevent the fusion of autophagosomes and lysosomes, thus increasing the accumulation of LC3-II and p62 [18]. To further explore whether autophagy was induced by PEDV infection, IPEC-J2 cells were pretreated with chloroquine and infected with PEDV for 18 h (CQ + PEDV). As shown in Figure 3a, the level of LC3-II in the CQ + PEDV group was significantly increased compared to that in the control group, whereas p62 was significantly decreased in the CQ + PEDV group. Although the LC3-II levels were markedly increased in the chloroquine-treated group, p62 levels were not significantly increased, indicating that chloroquine played a role in inhibiting autophagy flux. In the CQ + PEDV group, both LC3-II and p62 levels were significantly higher than those in the chloroquine group. PEDV N protein levels in the CQ + PEDV group were similar to those in the PEDV group, indicating that PEDV proliferation was not affected by chloroquine. These results suggest that PEDV infection in IPEC-J2 cells causes complete autophagic flux.

To determine whether autophagy could regulate the replication of PEDV, IPEC-J2 cells were pretreated with insulin to inhibit autophagy and infected with PEDV for 18 h. The levels of LC3-II and PEDV N proteins were detected using Western blotting. The results are shown in Figure 3b. Compared with the PEDV-infected group, the level of LC3-II decreased in the insulin + PEDV group, indicating that autophagy was inhibited. The level of the PEDV N protein also significantly decreased, indicating that PEDV replication was inhibited. These results suggest that the inhibition of autophagy can inhibit the replication of PEDV in host cells.

To determine whether promoting autophagy can regulate the replication of PEDV in IPEC-J2 cells, we used rapamycin, an autophagy-promoting agent, to pretreat IPEC-J2 cells and then infected the cells with PEDV for 18 h. The protein levels of LC3-II and PEDV N were detected using Western blotting. The results are shown in Figure 3c. Compared with the PEDV-infected group, LC3-II levels in the rapamycin + PEDV group were significantly upregulated, indicating that autophagy was promoted. The level of PEDV N protein was significantly increased, indicating that PEDV replication was promoted.

The PEDV-induced autophagic flow was visually assessed using a GFP-mCherry-LC3 construct. The results are shown in Figure 3d. Green and red fluorescent signals were observed in IPEC-J2 cells in both the control and PEDV-infected groups. The green fluorescence signal in the PEDV group was weaker than that in the control group, indicating that PEDV infection could cause autophagosome production and generate autophagic flow.

### 3.4. Transcriptome Sequencing of PEDV-Infected IPEC-J2 Cells

**Figure 4:**
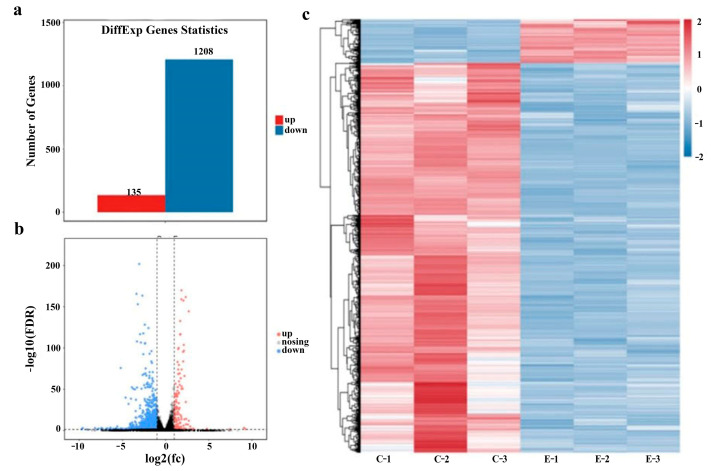
Statistics, volcano plot, and pattern clustering heat maps of differentially expressed mRNAs. (**a**) Statistical map of differential genes. (**b**) Volcano plot of differential gene comparison. (**c**) Cluster heat map of differential genes.

To screen for receptor proteins that induce autophagy in PEDV-infected IPEC-J2 host cells, RNA-seq technology was used to analyze the expression patterns of intracellular genes following the PEDV infection of IPEC-J2 cells. After screening and analysis, 21,022 mRNAs were identified in the control (non-infected) and PEDV-infected groups. Using DESeq2 software (version, 1.46.0) analysis, with |log2FC| ≥ 1 and a *p*-value < 0.05 as selection criteria, we screened 1343 differentially expressed mRNAs. The analysis showed that in PEDV-infected cells, the expression of 135 mRNAs increased significantly, and that of 1208 mRNAs was significantly lowered when compared to non-infected cells, as shown in Figure 4a.

A volcano plot was created based on the DESeq2 data, which shows the differential gene expression between the comparison groups (Figure 4b). In a volcano plot, upregulated genes are skewed to the right, and downregulated genes are skewed to the left, with the degree of statistical significance being indicated by the y value. Then, we carried out hierarchical clustering based on differences in gene expression patterns and created a heat map to render the clustering results, as shown in Figure 4c. In the heat map grid, each column represents a cell sample; each row represents a gene; and the level of gene expression is expressed in color where the redder the color is, the higher the expression, and the bluer the expression is, the lower the expression. According to cluster analysis, genes with similar gene expression profiles can be found. Genes with similar expression patterns may have common functions or participate in common metabolic and signaling pathways.

### 3.5. Analysis of Gene Ontology (GO) Enrichment

GO enrichment analysis was performed on the differentially expressed mRNAs, and the results are shown in Figure S1. The biological processes enriched were mainly related to cellular processes, metabolic processes, and biological regulation. In addition to biological processes, cellular components and molecular functions were included. The cell components were mainly concentrated in the cytosol and the membrane. Molecular functions were primarily related to binding and catalytic activity.

### 3.6. Analysis of Kyoto Encyclopedia of Genes and Genomes (KEGG) Enrichment

The KEGG pathway analysis was performed on the differentially expressed mRNAs, and the results are shown in Figure S2. The bubble chart shows the 20 pathways that were the most significantly enriched. Larger rich factor values indicate greater enrichment. The results show that the differentially expressed mRNAs in PEDV-infected IPEC-J2 cells were mainly enriched in steroid biosynthesis, terpenoid biosynthesis, Toll-like receptor signaling, viral proteins, cytokines, and cytokine receptors. Toll-like receptor 4 (TLR4) plays an important role in cell processes, the stimulation of the external environment, virus infection responses, intracellular material transport, and catabolism. TLR4 is closely related to AKT/mTOR signaling pathway conduction. This pathway regulates autophagy. The transferrin receptor (TFRC) plays a crucial role in cell processes, the stimulation of the external environment, phagosome generation, intracellular material transport and catabolism, and ferroptosis. TFRC is closely correlated with HIF-1 signaling. This pathway is closely related to intracellular hypoxic stress and the AKT signaling pathway. Gamma-aminobutyric acid type A receptor subunit gamma3 (GABRG3) is an important part of the cell membrane and is involved in the external stimulation of cells through the interactions between signal molecules. It is also a major component of the synaptic membrane. There have been few studies on GABRG3. Because the mRNA levels of these three genes were significantly increased in the sequencing results, TLR4, TFRC, and GABRG3 receptors were selected as candidate genes that may be involved in PEDV-induced autophagy.

### 3.7. TLR4 Knockdown Inhibited the Early Replication of PEDV

**Figure 5:**
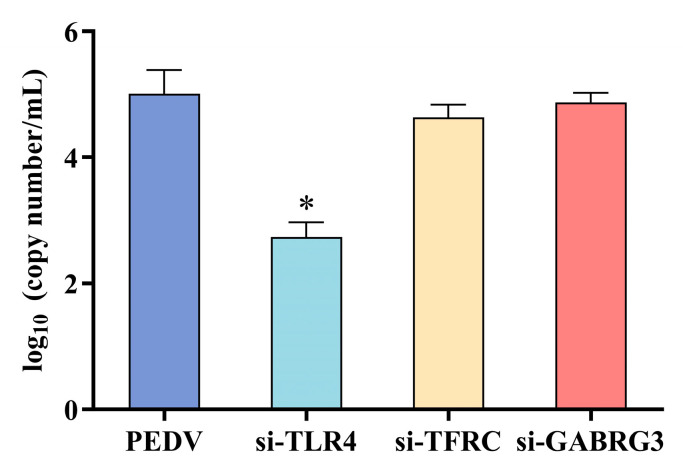
PEDV N gene expression in TLR4^−/−^, TFRC^−/−^, and GABRG3^−/−^ IPEC-J2 cells. * (*p* < 0.05).

The TLR4, TFRC, and GABRG3 genes in IPEC-J2 cells were knocked down using the small interfering RNA (siRNA) method. Cells were infected with PEDV for 18 h, and the level of PEDV N gene expression was detected using qRT-PCR to determine the impact on PEDV replication. As shown in Figure 5, TLR4^−/−^ in IPEC-J2 cells significantly inhibited PEDV replication, whereas the TFRC^−/−^ and GABRG3^−/−^ groups showed no significant inhibition in viral replication.

### 3.8. TLR4 Plays a Critical Role in PEDV Infection-Induced Autophagy

**Figure 6:**
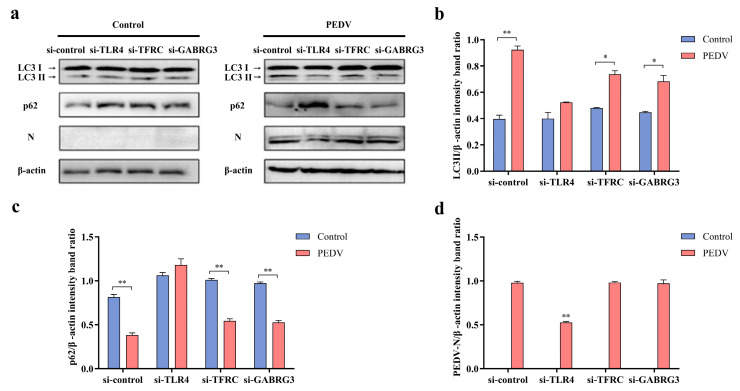
The effects of TLR4^−/−^, TFRC^−/−^, and GABRG3^−/−^ on autophagy, as assessed using Western blotting. (**a**) The changes in the protein expression of LC3-II, PEDV-N, and p62 in PEDV-J2-infected IPEC-J2 cells treated with siRNA detected using Western blotting. (**b**) The quantitative analysis of LC3-II and β-actin. (**c**) The quantitative analysis of p62 and β-actin. (**d**) The quantitative analysis of PEDV-N and β-actin. * (*p* < 0.05) and ** (*p* < 0.01).

To determine which receptor was related to PEDV infection-mediated autophagy in IPEC-J2 cells, siRNA was used to interfere with the expression of target genes in PEDV-infected IPEC-J2 cells. After 18 h, the levels of the autophagy marker protein LC3-II, autophagic flow protein p62, and PEDV N protein were detected using Western blotting and were analyzed comprehensively (Figure 6). Compared with the control group, the level of autophagy caused by PEDV infection was significantly reduced when TLR4 was knocked down, which manifested as a significant decrease in the expression of the LC3-II protein, while the level of p62 protein was not significantly decreased (Figure 6a–c). In contrast, when TFRC and GABRG3 were knocked down, the level of autophagy caused by PEDV infection was similar to that of the WT PEDV infection group, indicating that the knockdown of TFRC and GABRG3 did not significantly affect PEDV-induced autophagy. The PEDV N protein level in the TLR4 knockdown group was significantly lower than that in the WT PEDV infection group. There were no significant differences between the WT PEDV infection group and the TFRC and GABRG3 groups (Figure 6a,d). The above results demonstrate that the inhibition of TLR4 can inhibit PEDV infection-induced autophagy, indicating that TLR4 plays a key role in PEDV infection-induced autophagy.

### 3.9. Effect of TLR4 on AKT-mTOR Signaling Pathway

**Figure 7:**
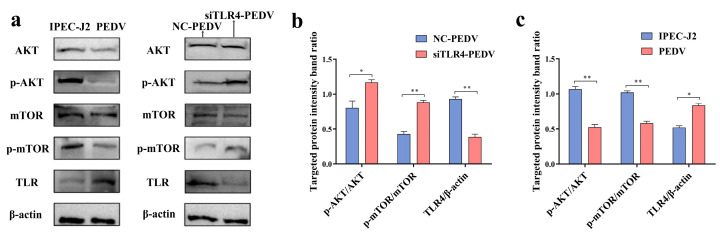
Analysis of AKT-mTOR phosphorylation using Western blotting. (**a**) The comparison of protein phosphorylation levels (AKT vs. p-AKT; mTOR vs. p-mTOR) and TLR4 expression using Western blotting in PEDV-infected cells in TLR4^−/−^ and WT cells. (**b**) The quantitative analysis of TLR4, β-actin, p-AKT, AKT, p-mTOR, and mTOR in TLR4^−/−^ IPEC-J2 cells. (**c**) The quantitative analysis of TLR4, β-actin, p-AKT, AKT, p-mTOR, and mTOR in WT IPEC-J2 cells. * (*p* < 0.05) and ** (*p* < 0.01).

The AKT-mTOR signaling pathway is crucially responsible for the conduction of autophagy-regulating signals. Studies have shown that the PEDV infection of IPEC-J2 cells induced autophagy through the AKT-mTOR signaling pathway. To verify the role of TLR4 in AKT-mTOR signaling, siRNA was used to inhibit the expression of TLR4 in PEDV-infected IPEC-J2 cells, and the activation of the AKT-mTOR signaling pathway was assessed. As shown in Figure 7, the phosphorylation of AKT-mTOR negatively regulated autophagy, and PEDV infection inhibited the phosphorylation of AKT-mTOR (Figure 7a,c). Compared to the WT PEDV-infected group, TLR4 knockdown resulted in the increased phosphorylation of AKT-mTOR (Figure 7a,b). The results described above indicate that PEDV-induced autophagy requires the stable expression of TLR4, and TLR4 inhibits the activity of the AKT-mTOR pathway to promote PEDV-induced autophagy.

### 3.10. Identification of Truncated Proteins That Induce Autophagy

**Figure 8:**
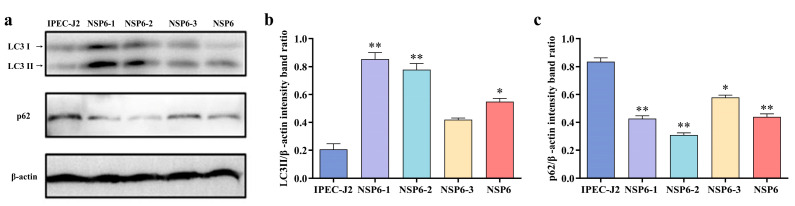
Analysis of LC3-II and p62 protein levels by Western blotting. (**a**) Changes in the expression of LC3-II and p62 in IPEC-J2 cells induced with NSP6 and its truncated proteins detected using Western blotting. (**b**) The quantitative analysis of LC3-II and β-actin. (**c**) The quantitative analysis of p62 and β-actin. * (*p* < 0.05) and ** (*p* < 0.01).

To analyze the key functional domains of NSP6-induced autophagy, the levels of LC3-II and p62 produced by truncated NSP6 proteins in IPEC-J2 cells were detected using Western blotting. As shown in Figure 8, compared with the control group, NSP6, NSP6-1, and NSP6-2 proteins significantly promoted LC3-II protein levels (Figure 8a,b), while p62 protein levels decreased (Figure 8a,c), indicating that NSP6, NSP6-1, and NSP6-2 significantly induce autophagy.

### 3.11. Analysis of Autophagic Flow Induced by Truncated NSP6 Proteins

**Figure 9:**
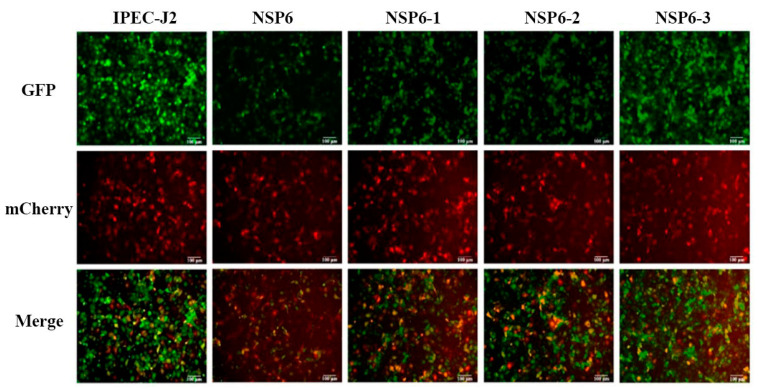
Autophagic flow was detected using GFP-mCherry-LC3 dual fluorescence labeling.

The double fluorescent labeling of GFP-mCherry-LC3 adenovirus was used to detect the autophagic flow induced by each truncated protein. As shown in Figure 9 (the scale is 100 μm), the green fluorescence signals of the NSP6-1 and NSP6-2 groups were weaker than those of the control and NSP6-3 groups. These results indicate that NSP6-1 and NSP6-2 could induce autophagic flow.

### 3.12. Identification of Functional Domains of NSP6 in Autophagy Induction

**Figure 10:**
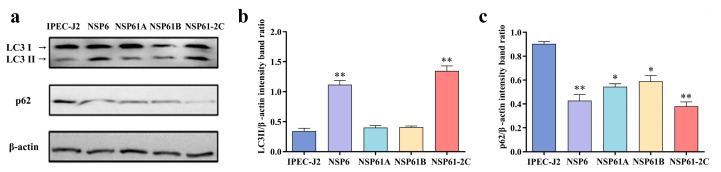
Analysis of LC3-II and p62 expression in IPEC-J2 cells using Western blotting. (**a**) Changes in the expression of LC3-II and p62 in IPEC-J2 cells induced with NSP61A, NSP61B, and NSP61-2C were detected using Western blotting. (**b**) The quantitative analysis of LC3-II and β-actin. (**c**) The quantitative analysis of p62 and β-actin. * (*p* < 0.05) and ** (*p* < 0.01).

To further identify the key functional domains of Nsp6 responsible for inducing autophagy in cells, we truncated Nsp6-1 and Nsp6-2 and constructed three eukaryotic expression vectors pCMV-Nsp61A, pCMV-Nsp61B, and pCMV-Nsp61-2C. Western blotting was used to detect changes in the LC3-II and p62 protein levels caused by the NSP6-1A, NSP6-1B, and NSP61-2C induction of IPEC-J2 cells to analyze the key functional domains of NSP6 in autophagy induction (Figure 10a). As shown in Figure 10, compared with the control group, NSP6, and NSP61-2C significantly promoted LC3-II protein expression (Figure 10b), while p62 protein levels decreased (Figure 10c), indicating that the functional domain in the NSP6-C segment was a key factor in inducing autophagy.

### 3.13. Analysis of Autophagic Flow Induced by NSP61-2 Truncated Proteins

**Figure 11:**
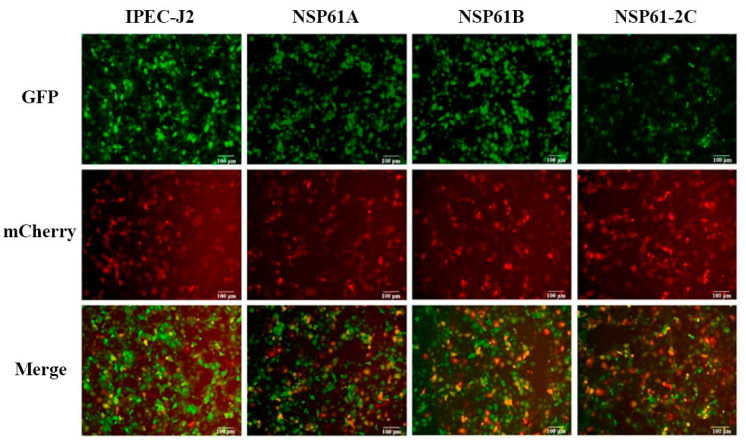
The autophagic flow was detected using GFP-mCherry-LC3 dual fluorescence labeling.

GFP-mCherry-LC3 was used to assess autophagic flow induced by the truncated proteins of NSP61-2. As shown in Figure 11 (the scale is 100 μm), the green fluorescent signal of the NSP61-2C group was weaker than those of the other groups. These results suggest that the NSP61-2C functional domain could significantly induce autophagic flow.

### 3.14. Effect of NSP61-2C on the AKT-mTOR Signaling Pathway

**Figure 12:**
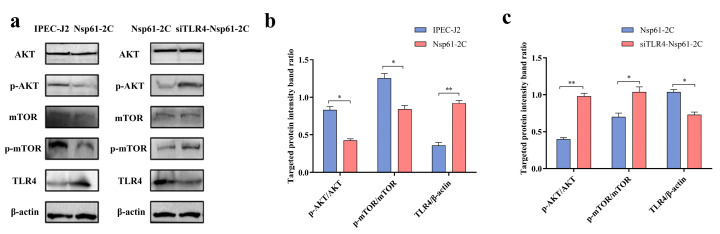
Analysis of AKT-mTOR signaling pathway activation using Western blotting. (**a**) Changes in the expression of AKT, p-AKT, mTOR, p-mTOR, and TLR4 proteins induced by NSP61-2C in TLR4^−/−^ and in WT IPEC-J2 cultures. (**b**) The quantitative analysis of p-AKT, AKT, p-mTOR, mTOR, TLR4, and β-actin in the NSP61-2C group. (**c**) The quantitative analysis of p-AKT, AKT, p-mTOR, mTOR, TLR4, and β-actin in the TLR4^−/−^ group. * (*p* < 0.05) and ** (*p* < 0.01).

To further explore the mechanism of NSP61-2C-mediated autophagy, siRNA was used to inhibit the expression of TLR4 in IPEC-J2 cells. Then, the cells were transfected with a pCMV-NSP61-2C eukaryotic expression plasmid, and the activation of the AKT-mTOR signaling pathway was assessed using Western blotting. As shown in Figure 12, AKT-mTOR phosphorylation was inhibited in the NSP61-2C transfected group (Figure 12a,c). Compared to the NSP61-2C group, TLR4^−/−^ cells demonstrated the increased phosphorylation of AKT and mTOR (Figure 12a,b). These results indicate that the stable expression of TLR4 is required for NSP61-2C-induced autophagy. In addition, NSP61-2C can negatively regulate the AKT-mTOR pathway through TLR4 to induce autophagy.

### 3.15. TLR4 and NSP61-2C Show Colocalization in Immunofluorescence Assays

**Figure 13:**
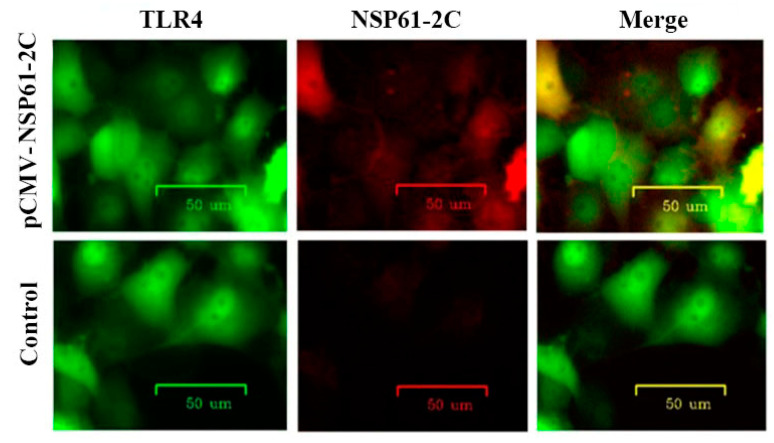
Detection of TLR4 and NSP61-2C co-localization using an indirect immunofluorescent assay.

To directly observe the effects of NSP61-2C and TLR4, indirect immunofluorescence assays were used to detect the colocalization of NSP61-2C and TLR4 in IPEC-J2 cells; the experimental results are shown in Figure 13. The eukaryotic expression of NSP61-2C (red) colocalized with the TLR4 receptor (green), and the fluorescence signal of NSP61-2C is distributed in the cell membrane, indicating copolymerization.

## 4. Discussion

Autophagy is an evolutionarily conserved cellular catabolic process that is required for normal cell function and also plays an important role in the antiviral and immune responses [25,44]. Viral infection and replication can induce autophagy [45,46]. Autophagic proteins can specifically sense microorganisms invading cells, such as viruses, and target them to the lysosomes for degradation [47]. Although autophagy can clear invading pathogenic microorganisms from host cells, an increasing body of research has revealed that numerous viruses have developed various strategies, including inhibiting, escaping, or manipulating the process of autophagy to achieve virus propagation in cells [48]. RNA viruses are the most common viruses that utilize autophagy to promote self-replication in host cells. These viruses use the energy and metabolic substances produced by autophagy for their own replication. Lipophagocytosis, a form of autophagy that degrades lipid droplets in cells, has also been found to be manipulated by viruses. After lipid droplets are taken up by cells, virus-induced autophagy can regulate the metabolism of cellular lipids and decompose lipid droplets into free fatty acids, leading to increased cellular β-oxidation and the production of large amounts of ATP, which can be used for viral replication [49].

Recently, some studies have found a correlation between PEDV infection and autophagy, but research has focused on identifying the PEDV protein responsible for autophagy induction, while the underlying mechanism of PEDV-induced autophagy has rarely been studied. In this study, TEM was used to observe the morphology of autophagosomes in bilayer vesicles 18 h after the PEDV infection of IPEC-J2 cells; the occurrence of PEDV-induced autophagy could be more directly detected through the observation of autophagosomes.

Transcriptomic analysis was carried out to assess differentially expressed genes under various conditions. This was carried out using GO and KEGG enrichment analyses. Transcriptomic analysis was performed on mRNAs of interest, and it was found that the differentially expressed genes were primarily related to steroid biosynthesis, terpenoid biosynthesis, Toll-like receptor signaling, viral proteins, cytokines, and cytokine receptors. Steroid biosynthesis plays a crucial role in regulating water–salt balance, metabolism, and stress responses, as well as initiating and maintaining sexual differentiation and reproduction [50]. The Toll-like receptor signaling pathway primarily functions in the innate immune response [51]. The NOD signaling pathway is involved in initial innate immune responses such as inflammation, cell damage, and stress, which means that PEDV infection can induce a series of metabolic reactions in cells, and the resulting cellular metabolites may be used for viral self-replication, which has properties similar to those of some viruses such as dengue virus [29].

PEDV-infected host cells produce a series of reactions that inhibit the proliferation of the virus, such as the production of cytokines. In addition, through GO and KEG analyses, we found that the TLR4 receptor was related to the AKT-mTOR signaling pathway, indicating that the TLR4 receptor may be a candidate gene involved in PEDV-induced autophagy. The AKT-mTOR signaling pathway is a popular topic in autophagy research. AKT phosphorylation can activate mTOR, which plays an important role in the regulation of autophagy. In addition, the mTOR signaling pathway is involved in a variety of intracellular pathological and physiological processes, such as mRNA signal transduction, cell cycle regulation, and apoptosis. The molecular structure of mTOR is relatively complex and it is capable of interacting with a number of proteins via different domains to perform, various biological functions and regulate diverse physiological processes [52]. There are two forms of mTOR: mTORC1 and mTORC2. The relationship between mTORC2 and autophagy is still unclear, although mTORC1 is a key inhibitory factor that induces autophagy and participates in the regulation of autophagy. In addition, mTORC1 is a target gene of rapamycin, which can act on the FRB domain of the mTOR protein to inhibit the activation of mTOR and, thus, induce autophagy [53]. In addition to mediating viral infection and host inflammation, TLR4 has also been found to be associated with autophagic processes. Some studies have shown that TLR4 can stimulate ubiquitin-specific protease 8 to mediate autophagy in SK-HEP-1 cells [54]. In addition to inducing autophagy, TLR4 can also induce the activation of inflammatory signaling pathways. Zhang et al. [55] found that S. typhimurium could mediate autophagy and inflammatory responses by activating the TLR4, MAPK, and NF-κB signaling pathways. In this study, transcriptome sequencing analysis showed that the mRNA level of TLR4 significantly increased in IPEC-J2 cells infected with PEDV, indicating that PEDV plays a regulatory role in the expression of TLR4 receptors during replication. When the expression of TLR4 was knocked down by siRNA, the protein levels of autophagy marker proteins, LC3-II and PEDV-N, were significantly decreased, and the phosphorylation level of AKT-mTOR was significantly increased, indicating that autophagy was inhibited and that the replication of PEDV was affected. These results indicate that TLR4 plays a crucial role in PEDV-induced autophagy in IPEC-J2 cells.

**Figure 14:**
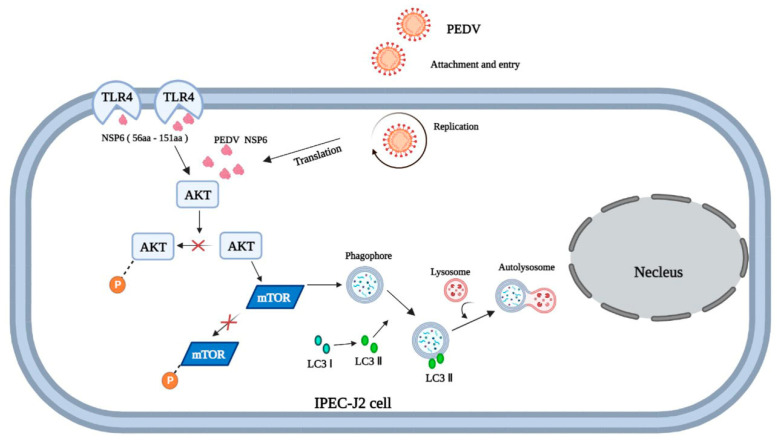
Molecular mechanism model diagram of autophagy induced by PEDV. **×**: represents the phosphorylation of AKT-mTOR is inhibited. The curved arrow represents the fusion of lysosomes and phagophore (autophagosomes).

Previous studies on autophagy induced by the non-structural protein NSP6 of coronaviruses found that this protein was capable of inducing autophagy during IBV and SARS-CoV-2 infection [8,56,57]. Studies on PEDV protein-induced autophagy have confirmed that PEDV NSP6 is a key protein in autophagy induction in IPEC-J2 cells [12], which was also confirmed in this study. By constructing the NSP6 eukaryotic expression plasmid and transfecting it into IPEC-J2 cells, the level of the autophagy marker LC3-II was found to be significantly increased using Western blot analysis. However, there are a few reports on which the functional domain of the NSP6 protein causes autophagy. To determine the key functional domain of the NSP6 protein that induces autophagy, we predicted potential functional domains using the SMART website and tried not to destroy its original domain. A eukaryotic expression plasmid with the truncated NSP6 protein was constructed and transfected into IPEC-J2 cells. The levels of LC3 II and p62 were determined using Western blotting, and the autophagosome and autophagy flux were analyzed using MDC fluorescence staining and GFP-mCherry double fluorescence labeling. The results show that the NSP61-2C segment (56-151aa) could significantly induce autophagy and inhibit the phosphorylation of AKT-mTOR through TLR4 to mediate autophagy (Figure 14).

Our findings highlight that PEDV induced autophagy through a mechanism involving the cellular pathogen receptor TLR4 and the AKT-mTOR-dependent pathway, and the viral protein NSP61-2C (56-151aa) to TLR4 ultimately induced autophagy and inactivated the AKT-mTOR pathway, which is an essential step for PEDV infection. This knowledge lays a foundation for future research to explore therapeutic strategies that target TLR4 or NSP6-associated autophagic pathways. Developing inhibitors that disrupt this interaction may offer a novel antiviral approach, potentially limiting PEDV replication and disease severity. Additionally, further investigation into the interplay between viral proteins and autophagic pathways could broaden our understanding of how PEDV, as well as other coronaviruses, exploit host cellular processes, potentially revealing targets for broad-spectrum antiviral therapies.

## Figures and Tables

**Table 1:** Primer sequences of PEDV-N.

Target Gene	Sequence (5′-3′)
β-actin	F: GGTGGGTATGGGTCAGAAAG R: TCCATGTCGTCCCAGTTGGT
PEDV-N	F: GGTATTGGAGAAAATCCTGACAGGGCAACAGCA R: GACGCATCAACACCTTTTTCGTTCCGCATC

**Table 2:** Sequences of siRNA.

Target Gene	Sequence (5′-3′)
Negative control	F: UUCUCCGAACGUGUCACGUTT R: ACGUGACACGUUCGGAGAATT
TFRC	F: GCAAUUGGUGUCUUGAUAUTT R: AUAUCAAGACACCAAUUGCTT
TLR4	F: GCAAAUGCCUCUGUGAUUUTT R: AAAUCACAGAGGCAUUUGCTT
GABRG3	F: GCUCCUCAGAAUUUGGAAUTT R: AUUCCAAAUUCUGAGGAGCTT

## Data Availability

The original contributions presented in the study are included in this article. Further inquiries can be directed to the corresponding authors.

## Supplementary Materials

The following supporting information can be downloaded at https://www.mdpi.com/article/10.3390/v16111787/s1. Figure S1. GO enrichment analysis of the differentially expressed mRNAs; Figure S2. KEGG pathway analysis of the differentially expressed mRNAs.
