# Supplementary figures and images for: IPEC-J2 Autophagy Induced by TLR4 and NSP6 Interactions Facilitate Porcine Epidemic Diarrhea Virus Replication
Source: Viruses. 2024 Nov 17;16(11):1787. doi: 10.3390/v16111787 (PMC11598845; doi:10.3390/v16111787)

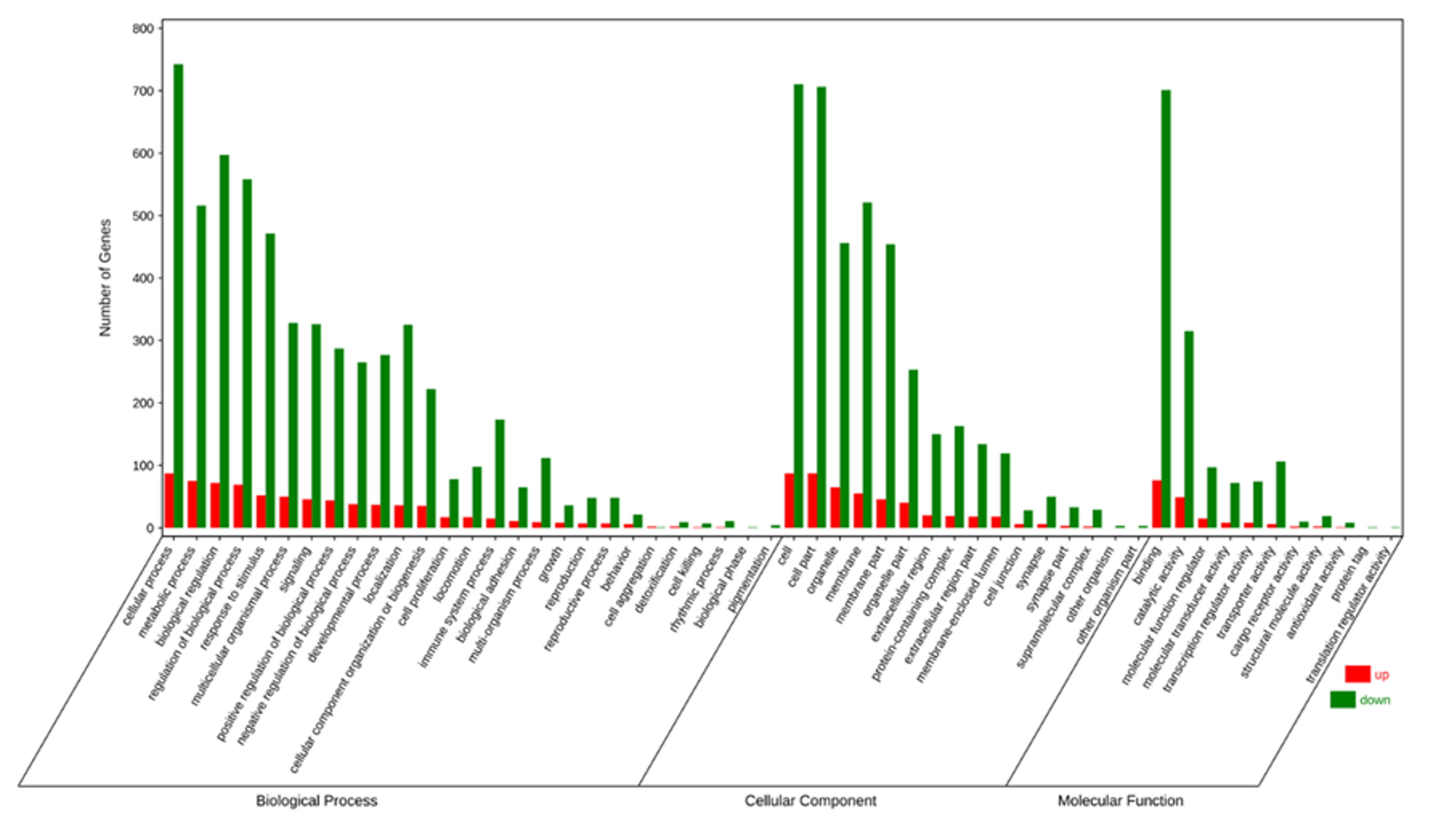

Supplement: Supplementary file 1 [file viruses-16-01787-s001.zip › FigureS1.jpg]

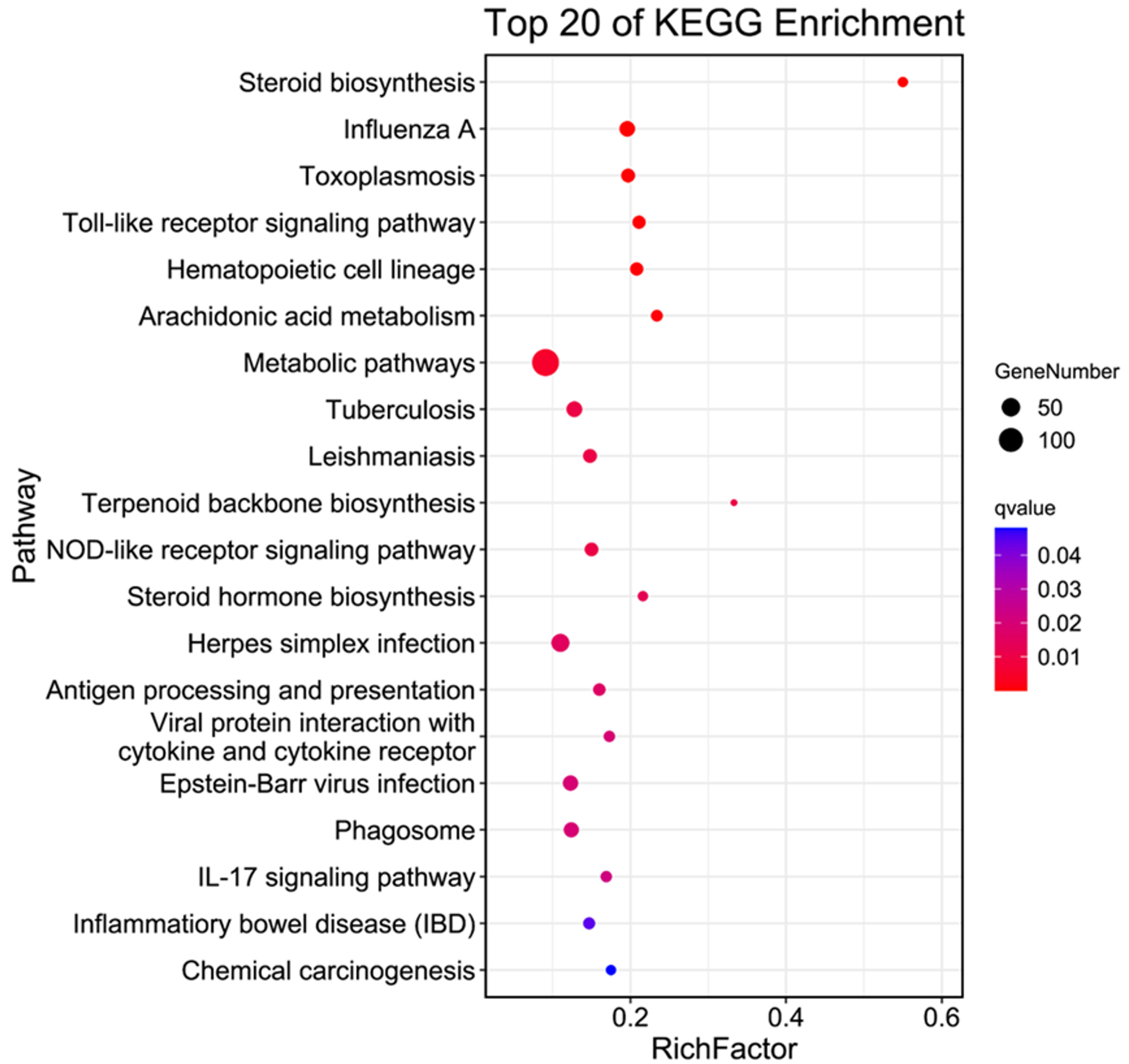

Supplement: Supplementary file 1 [file viruses-16-01787-s001.zip › FigureS2.jpg]
